# Clinical and molecular description of the first Italian cohort of 33 subjects with hypophosphatasia

**DOI:** 10.3389/fendo.2023.1205977

**Published:** 2023-08-01

**Authors:** Luigia Cinque, Flavia Pugliese, Antonio Stefano Salcuni, Domenico Trombetta, Claudia Battista, Tommaso Biagini, Bartolomeo Augello, Grazia Nardella, Francesco Conti, Sabrina Corbetta, Rita Fischetto, Thomas Foiadelli, Agostino Gaudio, Cosimo Giannini, Enrico Grosso, Gregorio Guabello, Stefania Massuras, Andrea Palermo, Luisa Politano, Francesca Pigliaru, Rosaria Maddalena Ruggeri, Emanuela Scarano, Piera Vicchio, Salvatore Cannavò, Mauro Celli, Francesco Petrizzelli, Mario Mastroianno, Marco Castori, Alfredo Scillitani, Vito Guarnieri

**Affiliations:** ^1^ Department of Pediatrics, “G D’Annunzioof Pediatrics, ” University of Chieti-Pescara, Foggia, Italy; ^2^ Unit of Endocrinology, Fondazione Istituto di Ricerca e Cura a Carattere Scientifico (IRCCS) Casa Sollievo della Sofferenza, Foggia, Italy; ^3^ Endocrinology and Metabolism Unit, University-Hospital S. Maria della Misericordia, Udine, Italy; ^4^ Laboratory of Oncology, Fondazione Istituto di Ricerca e Cura a Carattere Scientifico (IRCCS) Casa Sollievo della Sofferenza, Foggia, Italy; ^5^ Laboratory of Bioinformatics, Fondazione Istituto di Ricerca e Cura a Carattere Scientifico (IRCCS) Casa Sollievo della Sofferenza, San Giovanni Rotondo, Foggia, Italy; ^6^ Department of Clinical and Molecular Medicine, La Sapienza University, Rome, Italy; ^7^ Endocrinology and Diabetology Service, Istituto di Ricerca e Cura a Carattere Scientifico (IRCCS) Istituto Ortopedico Galeazzi, Milan, Italy; ^8^ Department of Biomedical, Surgical and Dental Sciences, University of Milan, Milan, Italy; ^9^ Clinical Genetics Unit, Department of Pediatric Medicine, Giovanni XXIII Children’s Hospital, Bari, Italy; ^10^ Pediatric Clinic, Department of Clinical-Surgical, Diagnostic and Pediatric Sciences, Istituto di Ricerca e Cura a Carattere Scientifico (IRCCS) Policlinico San Matteo Foundation-University of Pavia, Pavia, Italy; ^11^ Department of Clinical and Experimental Medicine, University of Catania, Catania, Italy; ^12^ Department of Pediatrics, “G D’Annunzio” University of Chieti-Pescara, Chieti, Italy; ^13^ Medical Genetics, Città della Salute e della Scienza University Hospital, Torino, Italy; ^14^ Reumatology Unit, Istituto di Ricerca e Cura a Carattere Scientifico (IRCCS) Istituto Ortopedico Galeazzi, Milan, Italy; ^15^ Unit of Endocrinology and Diabetes, Departmental Faculty of Medicine and Surgery, Campus Bio-Medico University of Rome, Rome, Italy; ^16^ Cardiomiology and Medical Genetics, University Hospital of Campania Luigi Vanvitelli, Naples, Italy; ^17^ Endocrine Unit, Azienda Ospedaliera-Universitaria of Cagliari, Cagliari, Italy; ^18^ Unit of Endocrinology, Department of Human Pathology DETEV “G. Barresi”, University of Messina, Messina, Italy; ^19^ Rare Diseases Unit, Department of Pediatrics, Istituto di Ricerca e Cura a Carattere Scientifico (IRCCS) Azienda Ospedaliero-Universitaria S. Orsola, Bologna, Bologna, Italy; ^20^ Department of Pediatrics, Jazzolino Hospital, Vibo Valentia, Italy; ^21^ Rare Bone Metabolism Center, Azienda Ospedaliera Universitaria (AOU) Policlinico Umberto I, Roma, Italy; ^22^ Scientific Direction, Fondazione Istituto di Ricerca e Cura a Carattere Scientifico (IRCCS) Casa Sollievo della Sofferenza, Foggia, Italy

**Keywords:** hypophosphatasia, alkaline phosphatase, genetics, dominant negative effect, ALPL, vitamin B6

## Abstract

**Introduction:**

Hypophosphatasia (HPP) is a rare genetic disease caused by inactivating variants of the ALPL gene. Few data are available on the clinical presentation in Italy and/or on Italian HPP surveys.

**Methods:**

There were 30 suspected HPP patients recruited from different Italian tertiary cares. Biological samples and related clinical, biochemical, and anamnestic data were collected and the ALPL gene sequenced. Search for large genomic deletions at the ALPL locus (1p36) was done. Phylogenetic conservation and modeling were applied to infer the effect of the variants on the protein structure.

**Results:**

There were 21 ALPL variants and one large genomic deletion found in 20 out of 30 patients. Unexpectedly, NGS-driven differential diagnosis allowed uncovering three hidden additional HPP cases, for a total of 33 HPP subjects. Eight out of 24 coding variants were novel and classified as “pathogenic”, “likely pathogenic”, and “variants of uncertain significance”. Bioinformatic analysis confirmed that all the variants strongly destabilize the homodimer structure. There were 10 cases with low ALP and high VitB6 that resulted negative to genetic testing, whereas two positive cases have an unexpected normal ALP value. No association was evident with other biochemical/clinical parameters.

**Discussion:**

We present the survey of HPP Italian patients with the highest ALPL mutation rate so far reported and confirm the complexity of a prompt recognition of the syndrome, mostly for HPP in adults. Low ALP and high VitB6 values are mandatory for the genetic screening, this latter remaining the gold standard not only to confirm the clinical diagnosis but also to make differential diagnosis, to identify carriers, to avoid likely dangerous therapy in unrecognized cases.

## Introduction

Hypophosphatasia (HPP, MIM #146300) is a rare genetic disorder affecting bone and teeth mineralization with multisystemic manifestations involving the nervous system, musculoskeletal apparatus, and kidneys (1). HPP is due to inactivating variants of the *ALPL* gene (NM_000478), encoding the TNSALP (tissue non-specific alkaline phosphatase), which determine a decrease up to the total loss of corresponding enzymatic activity (2).

While the primary manifestations of HPP are directly related to a deficient mineralization of bones and teeth (e.g., lack of or retarded ossification, fractures, bone deformities, caries, teeth fragility and loss), a plethora of additional manifestations, e.g., respiratory distress, muscle hypotonia, irritability, seizures, hypercalcemia/hypercalciuria, myalgias, arthralgias, and chondro- and nephrocalcinosis, define a spectrum of different severity depicted through the six phenotypic presentations, from the lethal “*in utero*” to the mildest form diagnosed in adults (1). Instead, the deficit of the TNSALP enzymatic activity causes the accumulation of three main corresponding substrates: pyrophosphate (PPi) in the extracellular spaces and pyridoxal-5′-phosphate (PLP) and phosphoethanolamine (PEA) in urine (3, 4). PPi is a strong inhibitor of the mineralization process, and its effect is paradigmatic of the primary HPP manifestations. At the neuronal level, in perinatal and infantile forms, HPP manifests with seizures due to the deficit of vitamin B6 (VitB6), which represent the dephosphorylated (i.e., active) PLP form (1). On the contrary, adult HPPs are characterized by mild symptoms such as stress fractures with delayed healing, periodontitis, and/or early loss of deciduous teeth and chondrocalcinosis due to calcium pyrophosphate deposition (1). With regard to PEA, whereas it is well known it accumulates in serum and urine, its specific metabolism is less evident as well as the effect of its accumulation on muscle function (4).

To date, more than 400 different deleterious (missense, nonsense, splicing, frameshift) ALPL variants have been identified, scattered throughout the 1,572-bp coding sequence (5), located at chromosome 1p36.12. Moreover, 14 large genomic deletions involving one or more exons or the whole genomic region encompassing the ALPL locus, have been reported (5), suggesting that large genomic deletions represent a less frequent mutational event of HPP.

HPP is transmitted in both autosomal dominant and autosomal recessive inheritances at the same locus. Variability is marked between and within families, especially in the autosomal dominant form, which is usually attributed to variants with the dominant negative effect (DNE, 6). In addition, the separation of inheritance pattern is not always clear-cut, with families ascertained by an index case with bi-allelic deleterious variants and minimal or mild manifestations in the “carrier” parents, who remained undiagnosed for decades. Inheritance pattern heterogeneity, the still high rate of variants of unknown significance (VUS) in ALPL and the marked variability in clinical expression make HPP a challenge for the health professionals (7).

Here, we present our experience of the last 5 years (March 2017–August 2022) with a cohort of 33 HPP patients recruited in different Italian tertiary clinical cares.

## Methods

### Patients

The Division of Medical Genetics, Fondazione IRCCS Casa Sollievo della Sofferenza, includes a regional referral center for hereditary bone metabolism disorders. The laboratory receives requests for genetic analysis from different Italian tertiary care centers. For the current study, cases were recruited from five pediatric (RF, TF, CG, ES, PV), three endocrinology (ASS, AG, RMM/SC), and four bone metabolism (FP/CB/AS, FC, SC/GG, MCe) referral centers for adults, two medical genetic (EG/SM, MC) units. From March 2017 to August 2022, 30 index individuals were enrolled for the study after preliminary biochemistry assessment confirmed the ALP value lower than the minimum range and measured at least twice and the VitB6 level higher than the maximum range (7–18 μg/l). Exclusion criteria were the assumption of drugs affecting bone turnover (i.e., bisphosphonates, denosumab) and/or diseases associated with low ALP activity/Vit B6 value (i.e., hypomagnesaemia, hypozincemia, celiac disease, Wilson disease, hypothyroidism, hypoparathyroidism, assumption of multivitamin supplements).

Three additional patients (#21, #22, and #23) underwent a next-generation sequencing (NGS) multigene panel analysis sequencing for hereditary disorders leading to early-onset osteoporosis/bone fragility and were included in the final survey after the identification of an ALPL variant: (i) subject #21 was a male newborn with fractures in the absence of trauma at 30 days of life with a suspicion of “shaken baby” syndrome; (ii) subject #22 was a boy of 2 years and 10 months, with an initial diagnosis of EDS/bone fragility; (iii) patient #23, was an adult man of 65 years, with persistent osteopenia and fractures. Unexpectedly, subjects (#21) and (#23) showed a normal (repeatedly) ALP value (**Table 1**).

**Table 1:** Summary of all the main clinical and molecular features of the patients recruited.

#ID	Sex	Age	AaO	AaD	Familial	Novel	Nucleotide	Predicted protein effect	PVS1 PM4	MAF (gnomAD)	PM2	PP2	Revel score	PP3	Domain^*^	PM1	PM5	PP5	ALP (UI)	Normal range (UI)	Vit B6 (7–18 ng/l)	PP4	Clinical interpretation
#1	F	45	20	41	NA	No	c.668G>A	p.(Arg223Gln)	/	1.6 × 10^-5^	Mod	Supp	0.97	Supp	CBS	Mod	Mod	Supp	21	35-107	44.4	Supp	LP
#2	F	64	37	58	NA	Yes	c.1415A>G	p.(His472Arg)	/	Absent	Mod	Supp	0.87	Supp	HI	Mod	/	/	8	40-150	33.5	Supp	LP
#3	F	75	66	71	NA	Yes	NA	del exon 2	vs	Absent	Mod	/	/	/	/	/	/	/	31	40-150	48.7	/	LP
#4	F	52	45	46	Yes	No	c.657G>T	p.(Met219Ile)	/	1.6 × 10^-5^	Mod	Supp	/	/	CBS	Mod	/	Supp	19	40-150	52.1	Supp	LP
#5	F	6	NA	15 months	Yes	No	c.571G>A c.963delG	p.(Glu191Lys) p.(Lys322Argfs*44)	/ vs	2.4 × 10^-3^ Absent	Mod Mod	Supp /	0.84 /	Supp /	AS B	Mod NA	Mod NA	vs Supp	20	156-369	383	Supp	LP P
#6	M	62	49	56	Yes	No	c.327C>A	p.(Asp109Glu)	/	Absent	Mod	Supp	0.89	Supp	AS	Mod	/	/	21	46-116	48.9	/	LP
#7	F	57	50	52	Yes	No	c.262G>A	p.(Glu88Lys)	/	Absent	Mod	Supp	0.77	Supp	HI	Mod	/	/	25	30-120	27	Supp	LP
#8	M	9	2.5	5	Yes	No	c.1172G>A	p.(Arg391His)	/	1.1 × 10^-5^	Mod	Supp	0.93	Supp	HI/CD	Mod	Supp	Supp	81	156-369	580	Supp	LP
#9	F	59	NA	54	NA	No	c.327C>A	p.(Asp109Glu)	/	Absent	Mod	Supp	0.89	Supp	AS	Mod	/	/	21	40-150	73.6	/	LP
#10	F	27	10	22	Yes	No Yes	c.407G>A c.870C>G	p.(Arg136His) p.(Phe290Leu)	/ /	1.2 × 10^-4^ Absent	Mod Mod	Supp Supp	0.7 0.82	Supp Supp	AS CBS	Supp Supp	Mod /	Supp Supp	18	40-150	82	Supp	LP VUS
#11	F	73	38	68	NA	Yes	c.1171insC	p.(Arg391Profs*14)	vs NA	4 × 10^-6^	Mod	/	/	/	HI/CD	/	NA	Supp	27	35-104	65.2	/	P
#12	F	38	30	31	NA	No	c.892G>A	p.(Glu298Lys)	/	2.1 × 10^-5^	Mod	Supp	0.93	Supp	CBS	Mod	/	/	25	40-150	21.4	Supp	LP
#13	F	57	NA	53	Yes	Yes	c.1573T>C	p.(*525Argextfs*11)	NA mod	4.1 × 10^-6^	Mod	/	/	/	GPI	/	/	/	41	45-117	23.6	Supp	VUS
#14	F	48	12	43	Yes	No	c.283G>A	p.(Val95Met)	/	3.3 × 10^-5^	Mod	Supp	0.76	Supp	AS	Mod	/	/	36	40-150	30.2	Supp	LP
#15	F	70	56	67	NA	Yes	c.800A>C	p.(His267Pro)	/	Absent	Mod	Supp	0.76	Supp	CBS	/	/	/	33	53-141	55	Supp	VUS
#16	M	68	63	65	NA	No	c.668G>A	p.(Arg223Gln)	/	1.6 × 10^-5^	Mod	Supp	0.96	Supp	CBS	Mod	/	/	41	45-117	45	Supp	LP
#17	F	37	14	35	NA	Yes	c.928dupT	p.(Ser310Profs*28)	vs NA	Absent	Mod	/	/	/	NA	/	/	/	24	33-98	88	Supp	LP
#18	F	51	NA	49	NA	Yes	c.250G>A	p.(Glu84Lys)	/	Absent	Mod	Supp	0.9	Supp	HI	Mod	/	/	19	42-98	235	/	LP
#19	F	61	17	60	NA	Yes	c.855C>G	p.(Tyr85*)	vs NA	Absent	Mod	/	/	/	HI	/	/	/	23	35-104	99.5	Supp	LP
#20	F	65	55	64	NA	No	c.98C>T	p.(Ala33Val)	/	1.4 × 10^-5^	Mod	Supp	0.87	Supp	N-ter α-helix	Supp	NA	Supp	38	45-117	46.4	Supp	LP
#21	M	5	20 ds	30 days	Yes	No	c.620A>C	p.(Gln207Pro)	/	Absent	Mod	Supp	0.98	Supp	NA	Mod	NA	Supp	147	90-345	/	/	LP
#22	M	5	1 y	2 years 10 months	Yes	No	c.542C>T	p.(Ser181Leu)	/	4 × 10^-5^	Mod	Supp	0.79	Supp	NA	Mod	Mod	Supp	129	141.8-336.4	/	/	LP
#23	M	66	10	65	NA	No	c.262G>A	p.(Glu88Lys)	/	Absent	Mod	Supp	0.77	Supp	HI	Mod	/	/	63	50-136	/	/	LP
#24	M	67	50	63	/	/	/	/	/	/	/	/	/	/	/	/	/	/	30	40-150	36.1	/	/
#25	M	70	50	68	/	/	/	/	/	/	/	/	/	/	/	/	/	/	28	30-120	47.9	/	/
#26	M	70	60	63	/	/	/	/	/	/	/	/	/	/	/	/	/	/	36	40-150	23.3	/	/
#27	M	81	NA	74	/	/	/	/	/	/	/	/	/	/	/	/	/	/	41	45-117	23.4	/	/
#28	F	91	70	86	/	/	/	/	/	/	/	/	/	/	/	/	/	/	36	40-150	58.5	/	/
#29	F	39	13	34	/	/	/	/	/	/	/	/	/	/	/	/	/	/	3,4	8.5-14.3	52.2	/	/
#30	M	62	40	59	/	/	/	/	/	/	/	/	/	/	/	/	/	/	39	40-150	32	/	/
#31	M	45	30	39	/	/	/	/	/	/	/	/	/	/	/	/	/	/	35	40-150	32	/	/
#32	M	65	53	59	/	/	/	/	/	/	/	/	/	/	/	/	/	/	38	40-150	33.5	/	/
#33	M	73	NA	69	/	/	/	/	/	/	/	/	/	/	/	/	/	/	25	30-120	25.3	/	/

For all the variants, pathogenicity criteria conferred by Franklin and the final interpretation according to the ACCMG guidelines were given: for some variants, the final interpretation switched from VUS to LP or from LP to P, in the presence of the specific phenotype criteria (PP4), that was bestowed only for patients having the TNSALP lower and VitB6 higher, than the normal range. AaO = age at onset; AaD = age at diagnosis; * for each variant, the corresponding domain was obtained from ref (1), ref (3), and ref (8); AS, active site; B, basis; CBS, calcium binding site; CD, crown domain; GPI, glycosylphosphatidylinositol anchor; HI, homodimer interface; NA, not available; NP, not present; Mod, moderate; Supp, supporting; vs, very strong.

Totally, 48.4% of the subjects were from South Italy, 27.2% from the Islands (Sardinia and Sicily), and 24.4% from North-Middle Italy. The sex ratio was F/M = 1.72 and median age = 51.8 +/- 19.4 years. Clinical, biochemical, and anamnestic data were collected.

### Sequencing

DNA was extracted from blood withdrawal, and the classic Sanger method was performed on 25 (75.7%) out of the 33 patients: DNA was PCR amplified, and amplicons were purified (ExoSAP-IT, Affymetrix, Thermo Fisher) and sequenced (Big Dye Terminator Cycle Sequencing Kit v 1.1, ABI3130XL Sequencer, Thermo Fisher) for the 12 coding exons (**Supplementary Material 1** for primers and PCR cycling conditions).

### NGS

Eight patients (24.3%) were screened through a SureSelect gene panel (Agilent Technologies, USA) designed to capture known genes associated with calcium metabolism disorders and bone fragility, including *ANO5*, *ALPL*, *B3GALT6*, *B4GALT7*, *BMP1*, *COL1A1*, *COL1A2*, *CREB3L1*, *CRTAP*, *DKK1*, *EN1*, *FAM46A*, *FKBP10*, *FKBP14*, *IFITM5*, *LRP5*, *MBTPS2*, *P3H1*, *P4HB*, *PLOD1*, *PLOD2*, *PLOD3*, *PLS3*, *PPIB*, *SEC24D*, *SERPINF1*, *SERPINH1*, *SLC39A13*, *SPARC*, *SP7*, *TMEM38B*, *WNT1*, and *YY1AP1*.

Targeted fragments were sequenced on a MiSeq platform (Illumina, USA) with MiSeq Reagent kit V3 300-cycle flow cells. Data analysis was performed considering the frequency, impact on the encoded protein, conservation, and expression of variants using distinct tools [ANNOVAR (9), dbSNP (10), 1000 Genomes (11), ExAC (12)]. Selected variants were interpreted according to the American College of Medical Genetics and Genomics/Association for Molecular Pathology (ACMGG/AMP) (13). Variants were confirmed by Sanger sequencing and segregation investigation performed on available relatives.

### Search for large genomic deletion

MLPA Kit (P484-ALPL, MRC Holland) was initially applied on negative subjects and then enlarged to the whole cohort, and it was carried out following the manufacturer’s instructions.

### Phylogenetic analysis

The level of conservation of the protein sequence was evaluated for the novel missense variants. From the Ensembl Genome Browser (14), the TNSALP protein orthologous sequences of the following organisms were downloaded: *Homo*, *Gorilla*, *Pan*, *Mus*, *Bos*, *Xaenopus*, *Tetraodon*, *Danio*. Then, Clustalw (15) was used to create the multi-alignment.

### 3D modeling

Atomic coordinates of TNSALP protein were obtained through AlphaFold v.2.0 (16). We retrieved the ALPL protein sequence in FASTA format from the Ensembl Genome Browser (14) and submitted it to ColabFold (17), which could predict the complex folding using the specialized AlphaFold-multimer model.

Then, molecular dynamics (MD) simulation was employed to refine the obtained models. Both the monomer and TNSALP dimer were embedded into a simulation box, extending up to 12 Å, using the tleap module of AmberTools21 (18), and solvated with the TIP3P water model and an appropriate number of Na+ and Cl- counter ions. The Amber ff14SB force field was applied to amino acids. Each model was subjected to energy minimization using steepest descent, followed by conjugate gradient methods. Then, they were gradually heated and equilibrated for approximately 5 ns, by time steps of 1 fs. A 10-Å cutoff was used for non-bonded short-range interactions, whereas long-range electrostatics were treated with the particle-mesh Ewald method. Temperature and pressure were maintained at 300 K and 101.3 kPa, respectively, using the Langevin dynamics and Piston method (19).

Following these preliminary steps, we implemented the Gaussian-accelerated molecular dynamics (GaMD), an enhanced molecular dynamic sampling method that works by providing a harmonic boost potential, which follows a Gaussian distribution, to smooth the system potential energy surface, thus causing the decrease of local energy barriers and accelerating the transitions between low-energy states. The boost potential can be applied in a dual-boost scheme: (i) a dihedral potential boost and (ii) a total potential boost. Maximum, minimum, average, and standard deviation values of the system potential were obtained from an initial ~2 ns conventional simulation with no boost potential, followed by a further GaMD equilibration of ~50 ns in which the boost potential was updated every 1.6 ns, thus reaching the equilibrium. Thus, each system was simulated for 100 ns using a timestep of 2 fs.

Finally, the stability of multiple TNSALP variants, p.(Ser181Leu), p.(Tyr28Asp), p.(Val95Met), p.(His267Pro), p.(His472Arg), p.(Met219Ile), p.(Ala179Thr), p.(Ala33Val), p.(Arg223Gln), (p.Arg391His), p.(Asp109Glu), p.(Gln207Pro), p.(Glu235Gly), p.(Glu298Lys), p.(Glu84Lys), and p.(Glu88Lys), and the double mutant p.(Arg136His)-p.(Phe290Leu), was investigated thermodynamically through the BuildModel and Stability function implemented in the FoldX algorithm (20). It was run with standard parameters. The same workflow was employed to evaluate the stop loss p.(*525Argfs*11) but using ColabFold to model the structure of both the monomer and dimer containing an additional residue fragment.

### Statistics

The original values of ALP were measured for non-uniform ranges; therefore, to make them comparable, the “ALP percentage deviation” = [(lower ALP value (for the corresponding reference interval)) - ALP)/lower ALP value (for the corresponding reference interval) x 100] was calculated. Analysis was performed with R (packages: stats, ggplot2, plyr, dbplyr, dplyr) (21–25).

## Results

### Clinical spectrum

Apart from early-onset cases or the ones that were resolved through the NGS-differential diagnosis (#21, #22 and #23), the remaining patients were firstly admitted for clinical problems related to bone pain, myalgia, fractures, nephrolithiasis, or early-onset or post-menopausal osteoporosis screening. This biased sample selection influenced the overall clinical spectrum of the cohort; instead, around 48% of patients claimed osteoporosis/low bone mass whereas 27% showed fractures at different sites. However, it is important to highlight that the whole cohort was then enrolled only after the biochemistry showed low ALP and high VitB6 (**Table 2**).

**Table 2:** Available clinical symptoms of patients.

ID	AaD	Sex	Mutated	Clinical symptoms	BMD lumbar spine and femoral neck	T score lumbar spine and femoral neck	Z score lumbar spine and femoral neck	AaO first symptoms
#1	41	F	Yes	Cramps, muscle tear right calf, left iliac crest bone pain	NA	NA	NA	20 Bone pain
#2	58	F	Yes	Bone arthralgia at the rachis and limbs, osteoporosis, nephrolithiasis, blood hypertension	0.65-0.51	-3.1, -2-9	NA	37
#3	71	F	Yes	Rib, femur, metacarpal fractures, parodontopathy, osteoporosis	0.66 (FN)	-3.0 (FN)	-1.0 (FN)	46 Osteop
#4	46	F	Yes	Scaphoid, toe and malleolus fractures, osteoarticular pain	1.03-0.81	-1.4, -1.4	-1.2, -1.3	45 Bone pain
#5	15 mesi	F	Yes	Edentulia, failure to thrive, valgus knees	NA	NA	NA	15 months Valgus knees
#6	56	M	Yes	Wrist bilateral, tibia and ribs fractures, coxalgia, recurrent nephrolithiasis, hypercalciuria	NA	NA	NA	49 Fractures
#7	52	F	Yes	Osteoporosis, vertebral fractures	NA	-3.2, -1.0	NA	50 Fracture
#8	5	M	Yes	Early edentulia, dyspraxia and lack of coordination, convulsions	NA	NA	NA	Early childhood Dyspraxia
#9	54	F	Yes	Osteoporosis	0.85-0.64	-2.8, -2.5	-1.1, -1.3	49 Bone pain
#10	22	F	Yes	Diffuse bone and muscle pains, low bone mass	1.06-0.66	-1, -2.6	-0.5, -2.3	10 Bone pain
#11	68	F	Yes	Early edentulia, osteoporosis, T2, L2, L3 vertebral, right metatarsus and phalanx (stress) fractures	0.66 – 0.53	-3.0, -2.8	NA	38 Edentulia
#12	31	F	Yes	Muscle weakness, cramps, myalgia	NA	NA	NA	30 Myalgia
#13	53	F	Yes	Osteoporosis	0.87-0.68	-2.6, -2.4	-1.0, -1.3	None
#14	43	F	Yes	Osteoarticular pains, ribs fractures, low bone mass	0.7-0.6	-3.1, -2.2	-2.8, -1.8	12 Bone pain
#15	67	F	Yes	Osteoarticular pains, osteoporosis, recurrent dental decay, rizarthrosis	NA	-2.3, -3.1	NA	56 Bone pain
#16	65	M	Yes	Osteoporosis, nephrolithiasis	0.76-0.62	-3.8, -3.5	-3.0, -1.9	51 Nephrolithiasis
#17	35	F	Yes	Cramps	NA	NA	NA	14 Cramps
#18	49	F	Yes	familiarity for osteoporosis	-0.85, 1-085	0.9, 0.1	1.6, 0.9	None
#19	60	F	Yes	Early edentulia, osteoporosis, motor delay	0,59-0,50	-4.1, -3.1		17 Edentulia
#20	64	F	Yes	Malleolus, toe, ribs, femur (sx and dx), L3, L5 fractures, nephrolithiasis	0.89-0.57	-2.0, -3.4	0.10, -1.8	55 Fractures
#21^ab^	30 days	M	Yes	Bone pain, femur fracture	NA	NA	NA	After birth
#22^a^	2 years 10 months	M	Yes	Pectus excavatum, scoliosis	NA	NA	NA	After birth
#23^ab^	65	M	Yes	Nephrocalcinosis, osteoporosis, L3 fracture, deafness	NA	-2.5, -2.0	-1.6, -1.6	10 Nephro
#24	63	M	No	Bone and muscle pain	NA	NA	NA	50 Bone pain
#25	68	M	No	Osteopenia, two vertebral fractures	NA	-0.9, -1.1	NA	50 Fracture
#26	63	M	No	Limbs muscle pain, blood hypertension, COPD	NA	NA	NA	60 Limbs pain
#27	74	M	No	Cardiomyopathy, nephrolithiasis	NA	NA	NA	NA
#28	86	F	No	Osteoporosis, osteoarticular pain	0.92-0.61	-2.2, -3.0	0, -1.1	70 Osteoporosis
#29	34	F	No	Femur fracture (atypical), diffuse myalgia, failure to thrive, arthralgia, early edentulia, bone angiomas	NA	NA	NA	13 Myalgia
#30	59	M	No	Difficulty climbing stairs and walking uphill	NA	NA	NA	40
#31	39	M	No	fracture (L5), muscle and bone pain	1.029 – 0.814	-1.6, -2	-1.8, -1.8	31 Fracture
#32	59	M	No	Fractures (T7 and T12), osteoporosis, hypercalciuria, nephrolithiasis	0.854 – 0.721	-3.1, -2.7	-2.6, -2.6	51 Osteoporosis
#33	69	M	No	T7–T8 vertebral fractures, hypercalciuria, odontopathy, renal microlithiasis	NA	-3.1, -2.7	NA	NA

a: Subjects with normal ALPL value; b: subjects identified through the NGS differential diagnosis; AaO, age at onset; COPD, chronic obstructive pulmonary disease; NA, not available; unless specified, all the fractures are due to “fragility,” whereas one case is reported as “atypical” or due to “stress”.

### Genetics

**Figure 1:**
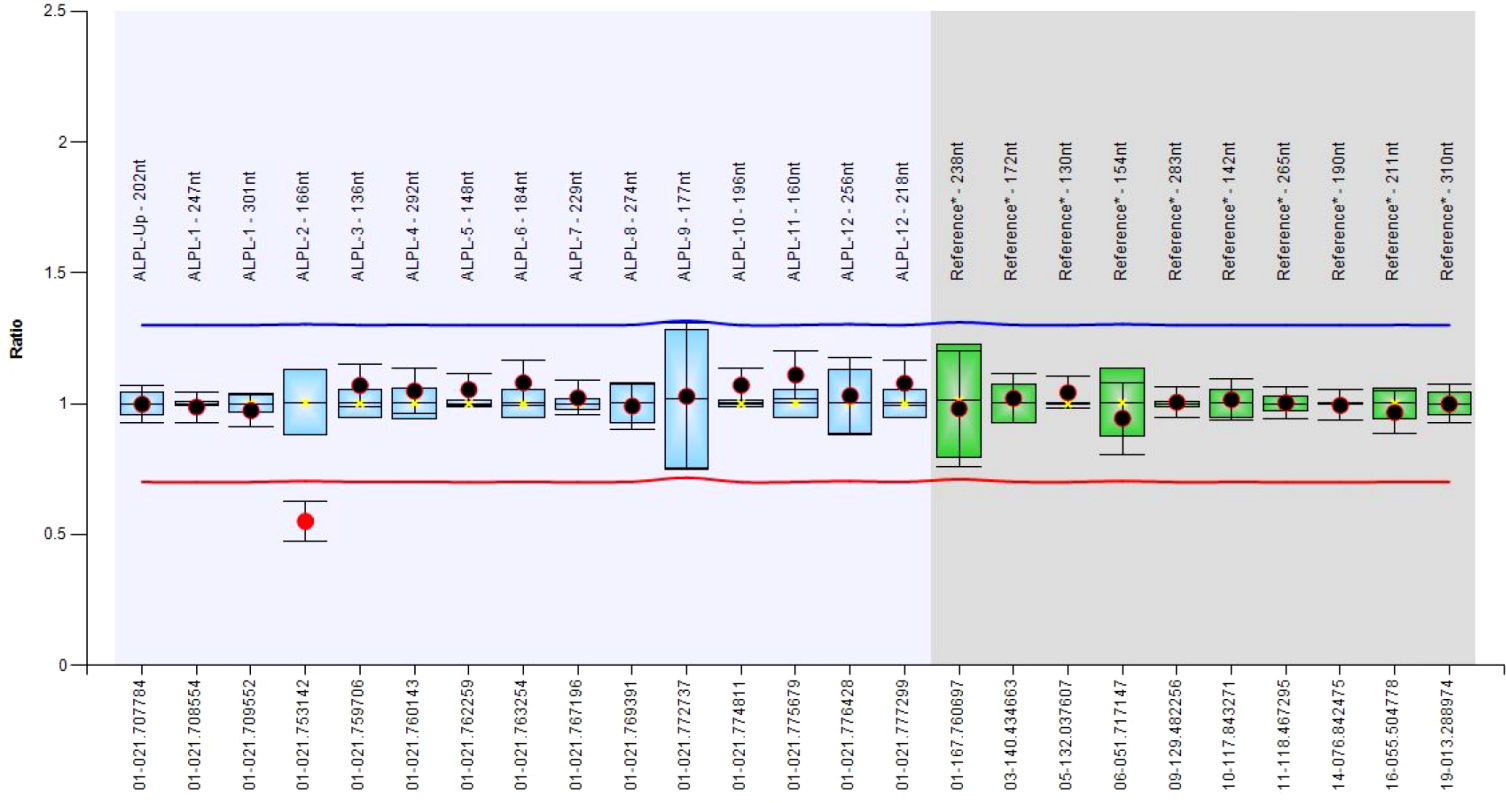
Boxplot chart showing the deletion of the exon 2.

From a total of 30 index individuals, we identified one or two single-nucleotide variants in 19 and a single-exon deletion (exon 2, **Figure 1**) in an additional individual, for a total of 20 cases (66.7%). To this cohort, we added three additional cases (#21-23), who received the diagnosis of HPP after the NGS multigene panel testing identified a likely pathogenic *ALPL* variant. Totally, 21 cases were heterozygous and two resultant compounds were heterozygous, a finding compatible with autosomal recessive inheritance (cases #5 and #10). Of the 25 identified mutated alleles (i.e., 21 heterozygous *plus* 2 compound heterozygous), we identified 22 different variants (21 single-nucleotide variants and 1, exon 2, deletion). Accordingly, 18 were private whereas 3 (i.e., c.262G>A, c.327C>A, and c.668G>A) recurred in 2 apparently unrelated pedigrees/index individuals. The 21 single occurrences were distributed as follows: 16 were missense, 3 frameshift, with the presumed insertion of a premature stop codon, 1 stop loss with the presumed insertion of 11 additional residues, 1 stop gain. Eight variants out of 21 (38%) were novel since they were not reported in the official database (5) (last release, March 2023). The Franklin online genomic variant interpretation tool (26) was used to determine the pathogenicity level of the eight novel changes, which resulted in one pathogenic, five likely pathogenic, and two VUS, following the ACMGG guidelines (13) (**Table 1**).

**Figure 2:**
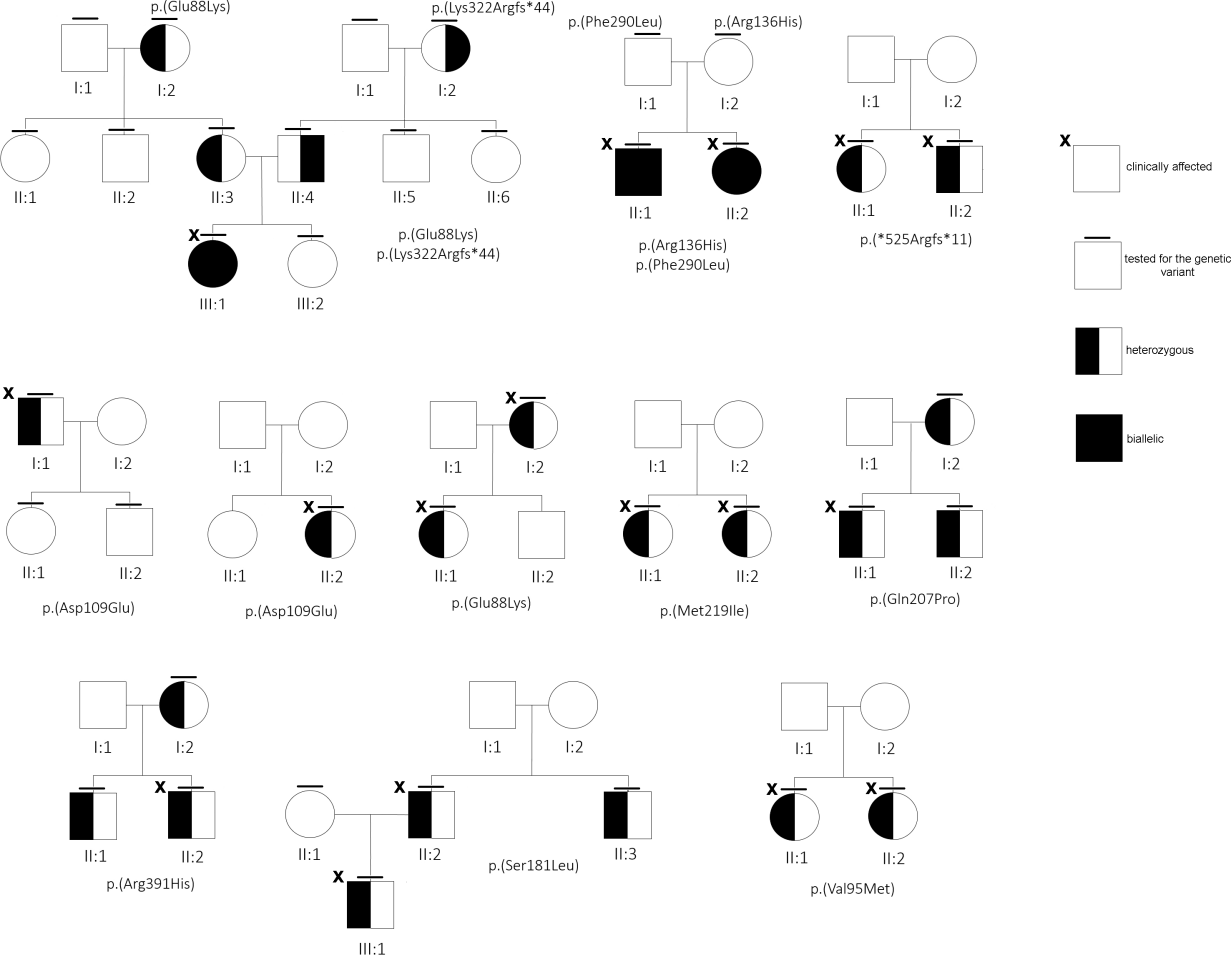
Family trees of some of the families under study.

There were 12 out of 23 mutated cases (52%) found to be familial, although for others, a clinical in-depth study or segregation analysis was not available, or yet concluded at the time of this report (**Figure 2**). Interestingly, 10 (out 30, 33.3%) subjects resulted to be negative, although at least for one case (#29) familiarity for low ALP value and clinical symptoms was reported.

### Biochemistry

**Figure 3:**
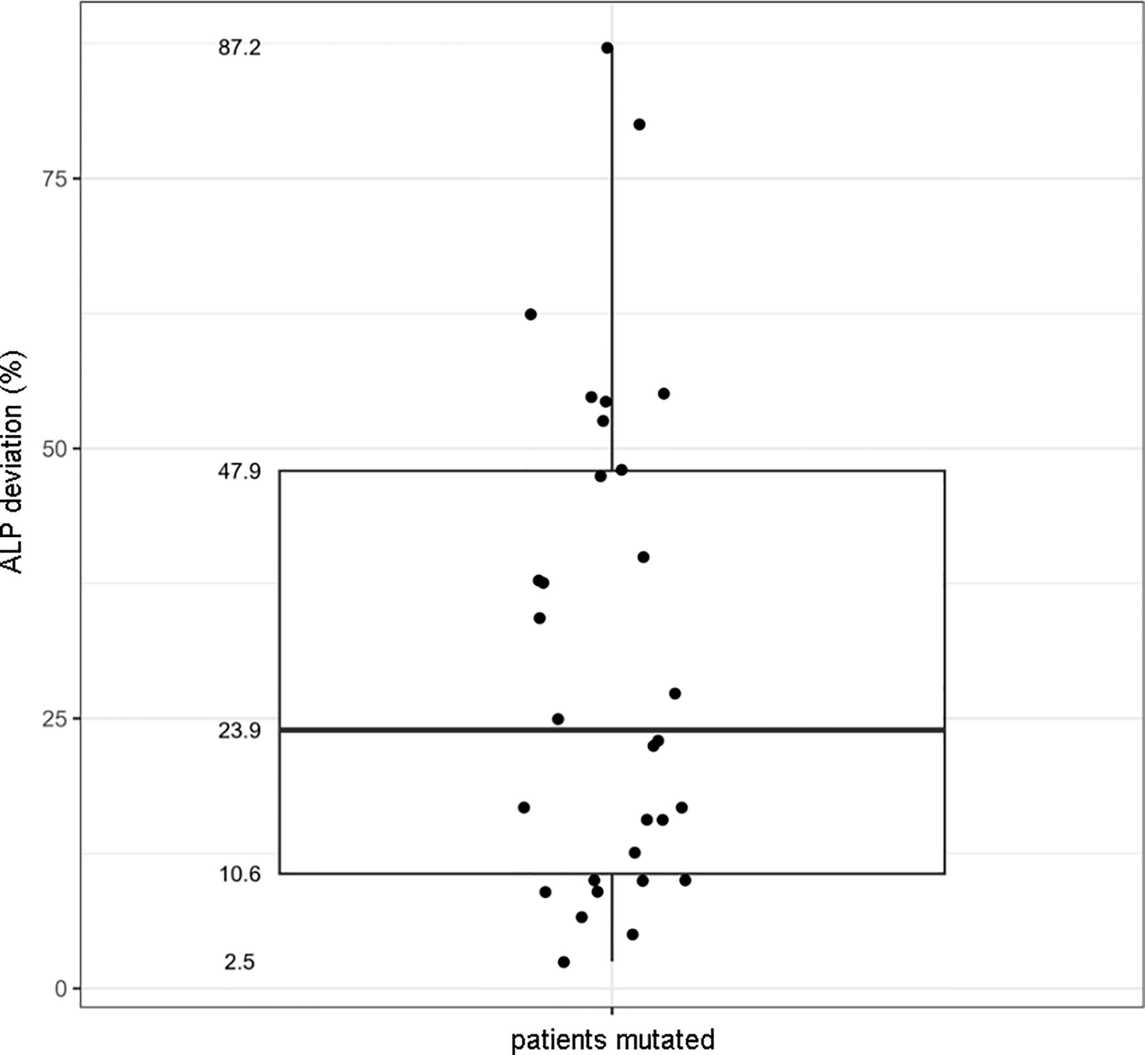
Boxplot showing the correlation between the presence of the ALPL mutation and the percentage deviation of the ALP value to the minimum range.

Due to the different assays and normal ranges used in the tertiary cares, we decided to plot the percentage deviation of the ALP value from the minimum value versus the presence/absence of the ALPL mutation. The correlation between the presence of a deleterious ALPL variant and the corresponding ALP serum value was, then, resolved in the boxplot in **Figure 3**: mutated patients showed an ALP value that was from 11 up to 49% lower than the minimum.

Other biochemical parameters (Ca, PTH, P, VitD, and Cr) were collected, but no association was found with the presence/absence of an ALPL variant, nor with the ALP value.

### Other genetic variants

Several SNPs were found among positive and negative patients (**Supplementary Material 2**). p.Arg152His is defined in ClinVar (8) as benign, with a MAF = 1.5%, in gnomAD. However, in our cohort, it was found in three subjects (9%), two of them carrying a pathogenic variant, with an overall frequency ~6 times higher than expected (**Supplementary Material 2**).

### Determination of the sequence conservation level

**Figure 4:**
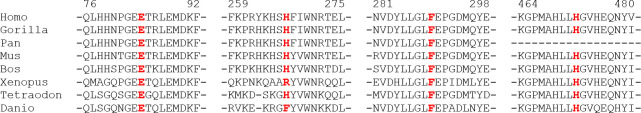
Phylogenetic multialignment showing the conservation level of the aminoadic sequence surrounding the novel changes, in different orthologous. Residues mutated are in red and show a high conservation up to Fishes.

The multi-alignment of the Alpl ortholog clearly showed that the novel missense variants affect residues conserved up to the amphibians, or up to the fishes, strongly supporting that they could have a deleterious effect on the protein function (**Figure 4**).

### Bioinformatic assessment

**Figure 5:**
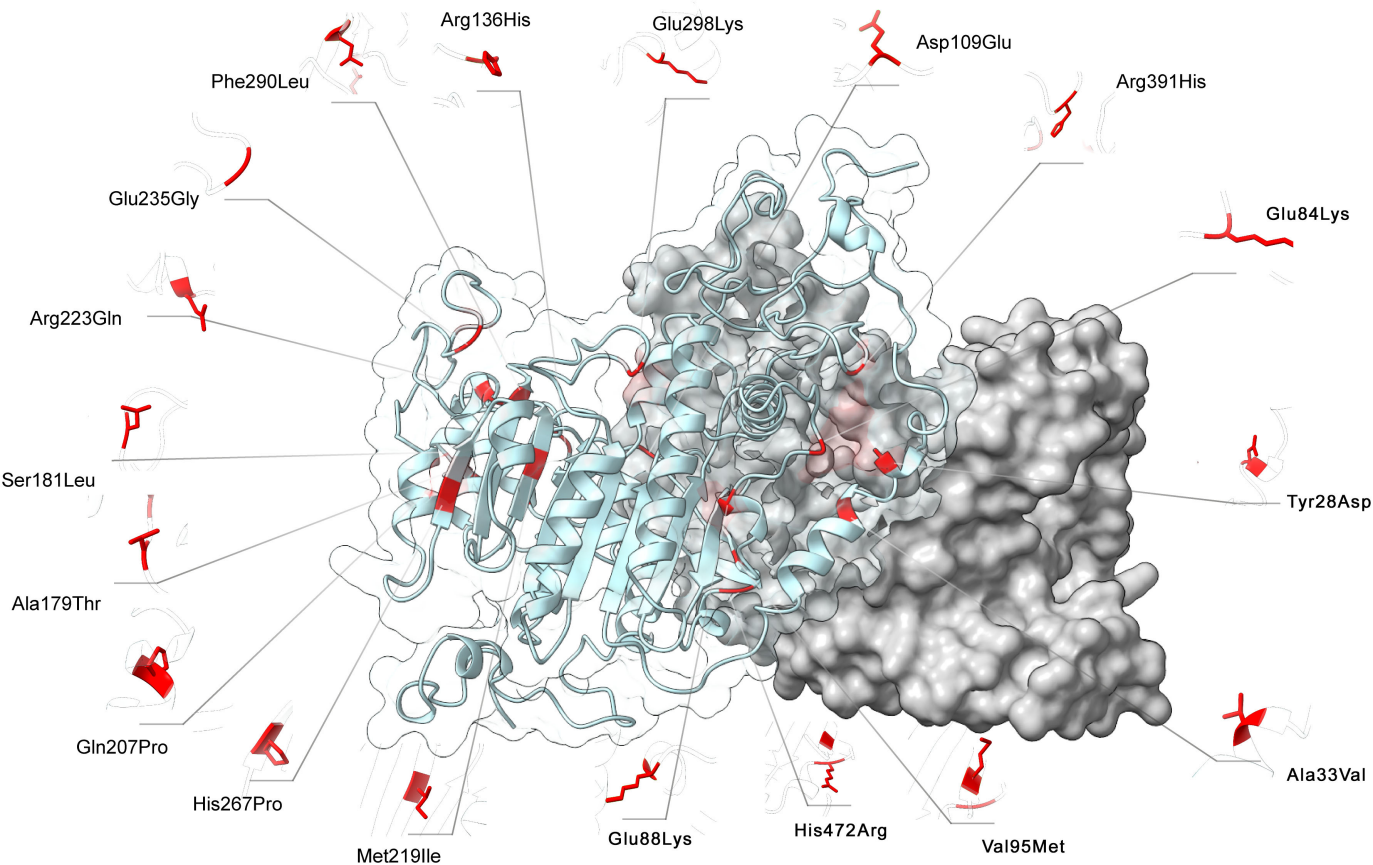
3D structure of ALPL dimer obtained through molecular modeling. Mutations were mapped on the wild-type structure and highlighted in red.

FoldX computed the total energy of ALPL dimers, wild-type and mutated, as a proxy of their overall stability and the van der Waals inter-residue clashes, as energy penalization factors. The free energy calculations are reported in **Table 3** and **Figure 5**. The difference in free-energy ΔΔG (ΔGmt – ΔGwt) for the majority of the ALPL amino acid changes was in the range to classify them as destabilizing whether only one or both monomers in the dimeric complex were mutated. In detail, p.(Asp109Glu), p.(His472Arg), p.(Met219Ile), p.(Ser181Leu), p.(Ala179Thr), p.(Arg391His), p.(Glu84Lys), and p.(Tyr28Asp) were observed as destabilizing whereas p.(Gln207Pro), p.(His267Pro), p.(Ala33Val), and p.(Arg223Gln) and the double mutant p.(Arg136His)-p.(Phe290Leu) could be classified as highly destabilizing. Conversely, p.(Glu88Lys) could be classified as stabilizing.

**Table 3:** ΔΔG (ΔGmt – Δgwt) for ALPL mutant protein.

Nucleotide	Protein	ΔΔG_single_ (kcal/mol)	ΔΔG_double_ (kcal/mol)
c.668G>A	p.(Arg223Gln)	5.52	12.15
c.657G>T	p.(Met219Ile)	2.5	4.64
c.262G>A	p.(Glu88Lys)	-0.85	-1.86
c.620A>C	p.(Gln207Pro)	5.31	8.74
c.892G>A	p.(Glu298Lys)	-0.95	-0.3
c.800A>C	p.(His267Pro)	4.45	9.1
c.542C>T	p.(Ser181Leu)	2.79	2.5
c.535G>A^†^	p.(Ala179Thr)	2.17	5.08
c.704A>G^†^	p.(Glu235Gly)	0.29	0.4
c.327C>A	p.(Asp109Glu)	2.12	4.87
c.1415A>G	p.(His472Arg)	0.64	4.33
c.1172G>A	p.(Arg391His)	1.5	4.07
c.283G>A	p.(Val95Met)	0.96	-1.5
c.250G>A	p.(Glu84Lys)	2.97	5.25
c.98C>T	p.(Ala33Val)	8.63	14.19
c.82T>G^†^	p.(Tyr28Asp)	2.96	5.98
***c.407G>A c.870G>C	***p.(Arg136His) p.(Phe290Leu)	7.15	7.55

ΔΔGsingle reports the stability of dimeric protein with only one monomer mutated, whereas in ΔΔGdouble, both the monomers were mutated. ***For the double mutant, we considered the case where both mutations were on the same monomer (ΔΔGsingle) and where each mutation was on a different monomer (ΔΔGdouble). ^†^These mutants were included as internal controls (see text).

p.(Glu298Lys) exhibited stabilizing effects when only one monomer was mutated; however, this alteration was compensated in the double-mutated dimer; on the contrary, p.(Val95Met) showed a destabilizing effect in the mutant monomer but a stabilizing effect with two mutations. Finally, p.(Glu235Gly) did not exhibit any structural impact on the ALPL protein.

A different approach was applied to the stop loss p.(*525Argfs*11), where the additional residue fragment in the protein structure could have a putative impact on the overall folding. Comparing the stability of both the monomer and the dimer with the wild type, we observed how the mutated protein was highly unstable in its monomeric form (ΔΔG = 555.75 kcal/mol), whereas the dimeric configuration was slightly more stable than the wild-type dimer (ΔΔG = -126.52 kcal/mol). It has to be noted that three mutants (p. (Tyr28Asp), p.(Ala179Thr), and p.(Glu235Gly)) were not part of the mutant collection found in our cohort, but they were previously studied (27, 28) and here included as internal controls.

## Discussion

HPP is a genetic disorder of bone metabolism featured by a variegated clinical presentation. Although the disease is caused by the lack of enzymatic TNSALP activity, symptoms often do not correlate with the severity of the enzymatic loss. The extreme variability of the resulting phenotype, ranging from lethal forms “*in utero*” to the osteoporotic type in adults, makes the condition hard to be promptly recognized (7, 29). This is mostly true for the milder forms, whose risk is to be confused with postmenopausal/idiopathic osteoporosis and consequently treated with bone antiresorptive drugs (i.e., bisphosphonates) that only succeed in worsening the bone disease (30). It was recently reported that up to 0.5% of osteoporotic patients may have low ALP activity, in which 87% had a deleterious *ALPL* variant (31). Hence, considering HPP in a wide differential diagnosis of osteoporosis/low bone mass could be relevant for both therapeutic and counseling issues.

### Genetics

In our cohort of suspected 30 HPP cases, we observed a mutation rate of 66.7% (20/30), sensibly higher if compared with previous studies (43%, 47%) (32, 33). Such a difference might lay on heterogeneity of patients’ selection, technical variability, and genetic background. Instead, Tenorio et al. (32) recruited 83 subjects on the basis of their clinical manifestation and low ALP activity, but the PLP value was not considered. Conversely, the cohort of 72 patients reported by Jandl et al. (33) was selected by low ALP activity, higher PLP, and clinical manifestation but consisted of only adult subjects (41.3–68.5 years). In the first case, it can be argued that adding a higher PLP level as an additional inclusion criterion could improve the case selection and the mutation rate. To confirm this assumption, we used, as internal control, a different cohort of subjects with low ALP activity but with normal or (presumably normal) not provided VitB6 value. Screening of the ALPL gene on these subjects was negative (data not shown), indicating that although hypophosphatasemia (with normal VitB6 serum level) is a not infrequent biochemical issue, it would not be related to the TNSALP gene. Concerning the Jandl et al.’s study, it cannot be excluded that the older age of the sample size may have influenced the mutation rate, perhaps due to the overlap with general osteoporosis.

In our survey, 10 patients with low ALP and high VitB6 values, one also having familial hypophosphatasemia, resulted to be negative. With regard to the clinical picture of these patients, whether the phenotype could be softened or worsened by the presence of alleged pathogenic variants lying in genetic regions not covered by the screening (such as deep intronic regions or 5′ and 3′UTR) or compensatory mechanisms or the action of genetic modifiers is far from being elucidated. In this regard, we kindly point out that our NGS panel contained the whole genomic (intronic and regulatory regions) of the ALPL gene, in order to identify deep intronic splicing variants, which, however, had never been found.

### Biochemical parameters

Interestingly, patients’ enrollment was driven by biochemistry values, regardless of the clinical spectrum that, as reported in **Table 2**, was highly variable, ranging from low bone mass/bone pain or just myalgia and actually reflecting the paradigmatic different HPP clinical variability. Thus, our work can confirm the utility of biomarkers in case of blurred clinical presentation, since only the ALP and VitB6 values were confirmed as the most suggestive clues with a strong correlation (up to 65% of cases) with the presence of a genetic lesion (**Figure 3**). In our previous work, we also suggested the predictive value of PEA (34); however, this metabolite, often along with VitB6, is not included in the routinely biochemistry panel, and, then, no PEA data were available at the time of this report.

### Genotype–phenotype correlations

Literature data recorded the p.(Gln207Pro) variant once in a severe autosomal recessive perinatal form in compound heterozygosity with p.(Arg71Pro) in a baby girl who died 3 h after birth (2). Patient #21 carried the same 207P variant and was affected by an autosomal dominant perinatal (30 days of life) form featured by severe fractures. However, the mother and grandmother, both carriers of the same variant, had an asymptomatic form with borderline ALPL levels. The p.(Glu88Lys) variant was found once in compound heterozygosity with the p.(Pro37Leu) in an 11-month-old boy affected by nephrocalcinosis and alterations of the fontanelle (35). Both our patients #7 and #23 carried the 88Lys in heterozygosity and #23 had nephrocalcinosis, since he was 10 years old. Whether the 88Lys variant is specifically accountable of the nephrocalcinosis requires further studies. It should be noted that cases #21, #22, and #23 were not initially included in the survey, as ALP values for #21 and #23 were normal. However, the use of NGS in the differential diagnosis helped to clarify, in both, an uncovered HPP form.

Interestingly, 2 subjects, #6 and #9 carrying the same variant, the p.D109E, both came from Sardinia and, although they denied any apparent kinship, a possible ancestor effect cannot be excluded.

### Protein variants and modelling

About the eight novel amino acid changes, the ALPL database has two different records for residue Glu84 (#18): the first was 84Val found in a family with autosomal dominant odonto-hypophosphatasia also characterized by rickets-like signs (36). The second, 84Asp, was found as a compound heterozygote in a 6-year-old girl with a childhood form. Despite no functional test being done on these variants, the authors suggested that both variants at Glu84 would bear a DNE, since the residue is located at the homodimer interface (36): this assumption was confirmed by our bioinformatic investigation.

No data can be inferred from the literature about the effect of the 267Pro (#15) or 472Arg (#2) mutants. Conversely, with regard to the Phe290 variant, Mornet et al. proved that this residue, together with Glu235, Glu291, and Asp306, coordinates the binding of the calcium atoms (37): it can therefore be deduced that the 290Leu (#10) variant could severely impair the enzymatic activity. Our modeling study confirms that all three mutants destabilize the homodimer structure.

About the stop gain p.(Tyr85*) (#19) and both the frameshift mutations, p.(Ser310Profs*28) (#17) and p.(Arg391Profs*14) (#11), that, presumably, introduce a premature stop codon, the mutated proteins are likely to undergo proteasomal degradation (38), or, if translated, they are totally inactive, due the loss of (more than) 50% of the canonical protein structure. In contrast, p.(*525Argextfs*11) (#13) does not interrupt the coding frame, as it leads to correct translation of the whole protein with presumed 11 additional residues. Although in the absence of functional data, the effect of this supplemental tail could not be considered detrimental for the enzymatic activity; instead, the mutant does not seem to bear the DNE since the phenotype of patient #13 (and of her brother, **Figure 2**) overlaps with classical osteoporosis with a borderline-low ALP activity. In our computational modeling, the mutant homodimer p.(*525Argextfs*11) appears more stable than the monomer, so we cannot exclude that the destabilized structure is partially mitigated. This is in contrast with data reporting that a similar mutation, p.(Leu520Argfs*86), that adds more than 80 residues at the C-terminal tail was proven to lack GPI binding with a consequent severe reduced enzymatic activity (39).

### SNPs

There were 22 common intronic (nr 14) and exonic (nr 8) variants identified: 17 have a MAF in gnomAD > 0.05, whereas the remaining were rarer (**Supplementary Material 3**). No correlation was attempted with the presence of fractures, nor with biochemical values, as previously suggested (40, 41), since the distribution of the SNPs in the whole cohort (positive, negative, low, or normal ALP patients) was not different (data not shown).

p.(Arg152His) was found in three patients (out of 33, 9.3%) with a frequency six times higher than that reported in gnomAD (MAF = 1.5%): in two cases (#7 and #19), it was identified together with a pathogenic ALPL variant. Whether 152His could actually play the role of the phenotype modifier or it is in linkage disequilibrium with an actual pathogenic variant located in genetic regions not analyzed would be a hypothesis to shed light on. Moreover, it cannot be excluded that its frequency is higher in the Italian population, although there is no evidence in public databases.

## Conclusions

To the best of our knowledge, our genetic study represents the first reporting of a so high mutation rate in an Italian HPP cohort: it follows our previous work on a smaller survey (34) and describes a total of 23 molecularly confirmed HPP cases, 8 novel ALPL variants, 1 large ALPL deletion, and 1 case with familial hypophosphatasemia in which the search for the molecular basis has to date been unsuccessful. The identification of eight novel variants and the detailed clinical description increase the knowledge on the effect of ALPL pathogenic variants associated with this disease and provides a clear landscape about the spread and presentation of this disease in Italy.

We confirm the complexity of a timely recognition of the syndrome, mostly for adult forms, due the high clinical variability In this regard, the suspicious HPP can derive from the biochemical values, low ALP, and high VitB6, and not necessarily from the clinical phenotype that can be extremely variable. The clinical diagnosis must then be confirmed by the genetic analysis that, undoubtedly, represents an essential aid to identify carriers to address to the follow-up and to make the differential diagnosis, avoiding dangerous therapies in unrecognized cases.

## Data availability statement

The data presented in the study are deposited in the European Variation Archive (EVA) repository at the EMBL-EBI institute, with the following accessions: Project - PRJEB61880 and Analyses - ERZ18242459 (42).

## Ethics statement

The studies involving human participants were reviewed and approved by Ethics Committee at Fondazione IRCCS Casa Sollievo della Sofferenza (Prot 201/CE, Dec 2017). Written informed consent to participate in this study was provided by the participants’ legal guardian/next of kin.

## Author contributions

Conceptualization: LC, ASS, AS, and VG; methodology: LC, DT, BA, and GN; software: TB, FPe, and MM; validation: LC, FPu, and VG; formal analysis: FPu, ASS, and AS; clinical investigation: FPu, ASS, CB, FC, SCo, RF, TF, AG, CG, EG, GG, SM, AP, LP, FPi, RR, ES, PV, SCa, MCe, MCa, and AS; resources: MCa; data curation: LC; writing original draft preparation: AS and VG; writing, review, and editing: MCa, AS, and VG. All authors contributed to the article and approved the submitted version.
