# Supplementary material for: Clinical and molecular description of the first Italian cohort of 33 subjects with hypophosphatasia
Source: Front Endocrinol (Lausanne). 2023 Aug 1;14:1205977. doi: 10.3389/fendo.2023.1205977 (PMC10433156; doi:10.3389/fendo.2023.1205977)
Supplement: Supplementary file 1 [file Table_1.docx]

**Clinical and molecular description of the first Italian cohort of 33 subjects with Hypophosphatasia**

**Journal of Endocrinological Investigation**

| **Exon** | **Forward** | **Reverse** | **Tm** | **bp** |
| --- | --- | --- | --- | --- |
| Exon 2 | AATGGGACGGAACTGCTTTG | TGCCCTCATCATACCCCATC | 60 °C | 468 |
| Exon 3-4 | GGGGATCTGTACGTCTGGAG | CTCTGGCTGCTGTCATGTTC | 60 °C | 737 |
| Exon 5 | GTCCCCATGGTGTGAGTGTA | CCTTTTCTAGCCCCTTCCCA | 60 °C | 353 |
| Exon 6 | CTGTGGATGGGGAGACTGAG | CGCAGAGAAATCCCACAGTG | 60 °C | 502 |
| Exon 7 | TGGACAAGTAAGGCCCAGAG | AGAACTTCAGAGCAGGGGTC | 60 °C | 385 |
| Exon 8 | TCTTGAGGTCAGGGATGGTG | ATTCCAGGAACCAGAACCCC | 60 °C | 389 |
| Exon 9 | GCCACCATACTCTACCCCAA | CCCAAACCAGTCAGTTCCCT | 60 °C | 360 |
| Exon 10 | GAGCAGATCTTCCTCCCCTC | GTAACTCTTCCTCCCCACCC | 60 °C | 383 |
| Exon 11 | ATGAATGGGAGGGACATGGG | ATGCCCAAAGTCTCAGAGCT | 60 °C | 538 |
| Exon 12 | TCCCACATTGAGCCTCCTTT | GTGTGGGAAGTTGGCATCTG | 60 °C | 453 |

L Cinque, F Pugliese, AS Salcuni, D Trombetta, C Battista, T Biagini, B Augello, G Nardella, F Conti, S Corbetta, R Fischetto, T Foiadelli, A Gaudio, C Giannini, E Grosso, G Guabello, S Massuras, A Palermo, L Politano, F Pigliaru, RM Ruggeri, E Scarano, P Vicchio, S Cannavò, M Celli, F Petrizzelli, M Mastroianno, M Castori, A Scillitani, V Guarnieri

Division of Medical Genetics, Fondazione IRCCS Casa Sollievo della Sofferenza, 71013 Foggia, Italy; v.guarnieri@operapadrepio.it

Supplemental Material 1. Sequences of the primers, annealing temperatures and size of the amplicons.
