# Supplementary material for: Clinical and molecular description of the first Italian cohort of 33 subjects with hypophosphatasia
Source: Front Endocrinol (Lausanne). 2023 Aug 1;14:1205977. doi: 10.3389/fendo.2023.1205977 (PMC10433156; doi:10.3389/fendo.2023.1205977)
Supplement: Supplementary file 2 [file Table_2.docx]

**Clinical and molecular description of the first Italian cohort of 33 subjects with Hypophosphatasia**

**Journal of Endocrinological Investigation**

L Cinque, F Pugliese, AS Salcuni, D Trombetta, C Battista, T Biagini, B Augello, G Nardella, F Conti, S Corbetta, R Fischetto, T Foiadelli, A Gaudio, C Giannini, E Grosso, G Guabello, S Massuras, A Palermo, L Politano, F Pigliaru, RM Ruggeri, E Scarano, P Vicchio, S Cannavò, M Celli, F Petrizzelli, M Mastroianno, M Castori, A Scillitani, V Guarnieri

Division of Medical Genetics, Fondazione IRCCS Casa Sollievo della Sofferenza, 71013 Foggia, Italy; v.guarnieri@operapadrepio.it

| **ID** | **Mutated** | **Mg**  **(1.7 - 2.2 mg/dL)** | **Ca**  **(8.6 - 10.2 mg/dL)** | **PTH**  **(14 - 65 pg/mL)** | **Vit D > 30 ng/mL** | **Creat*** | **P**  **(2.8 to 4.5 mg/dL)** |
| --- | --- | --- | --- | --- | --- | --- | --- |
| #1 | Y | 2 | 10 | 16.9 | 44.3 | 0.81 | 3.7 |
| #2 | Y | 2.2 | 10.6 | 94.2 | 9.9 | 2 | 6 |
| #3 | Y |  | 10.1 | 22 | 36 |  |  |
| #4 | Y | 2.3 | 8.1 | 48.8 | 32.1 | 0.95 | 2.5 |
| #5 | Y |  |  |  |  |  |  |
| #6 | Y |  | 9.5 | 66 | 36 | 1.11 | 2.6 |
| #7 | Y |  | 9.2 | 45 | 32.2 | 0.98 | 4.5 |
| #8 | Y |  | 10.1 | 23 | 20 | 0.46 | 6.1 |
| #9 | Y |  | 8.8 | 39 | 21 | 0.77 | 3.3 |
| #10 | Y |  | 9.5 | 31.5 | 22.9 | 0.62 | 4.7 |
| #11 | Y |  | 9 | 51 | 13 | 0.65 | 3.9 |
| #12 | Y | 1.7 | 9.4 |  | 16.4 | 0.71 | 3.6 |
| #13 | Y |  | 9.3 | 64.8 | 44.1 | 0.8 | 3.7 |
| #14 | Y |  | 9 | 49.5 | 61.5 | 0.6 | 3.1 |
| #15 | Y |  | 9.2 |  | 44.5 | 0.85 | 3.7 |
| #16 | Y |  | 8.6 | 35.6 | 61.2 | 0.5 | 3 |
| #17 | Y | 0.74 | 9.44* | < 4* | 39.3 | 0.8 | 1.28 |
| #18 | Y |  | 9.5 |  | 39 | 0.97 |  |
| #19 | Y |  | 10.3 | 54.1 | 44.5 | 0.68 | 3.4 |
| #20 | Y | 2.1 | 8.54 | 69 | 38.1 | 0.5 | 3.3 |
| #21^†^ | Y |  | 10.8 | 27.8 | 26.3 |  |  |
| #22 | Y |  | 9.2 | 20.4 | 60.5 | 0.27 | 4.9 |
| #23^†^ | Y |  | 9.7 |  | 41.6 |  | 3.7 |
| #24 |  | 2.3 | 9.3 | 20.8 | 37.6 | 1 | 2.9 |
| #25 |  |  | 9.2 | 19 | 41 | 0.9 | 5 |
| #26 |  | 1.7 | 10.4 |  | 25 | 0.86 | 3.1 |
| #27 |  |  | 8.4 | 44.1 | 44.1 | 0.97 | 3.5 |
| #28 |  |  | 8.5 | 93.4 | 50.8 | 1.9 | 4.6 |
| #29 |  |  | 9.1 | 27 | 19 | 0.68 | 3.5 |
| #30 |  | 1.8 | 10 | 38.5 | 20 | 0.63 | 3.8 |
| #31 |  |  | 8.8 | 60 | 40 | 0.9 | 2.9 |
| #32 |  |  | 8.6 | 64.6 | 42.3 | 0.82 | 3 |
| #33 |  | 2.21 | 9.7 | 26 | 26 | 1.1 | 4.8 |

Supplemental Material 2. Other biochemical value of the cohort under study. *0.7 to 1.3 mg/dL (61.9 to 114.9 µmol/L) for men and 0.6 to 1.1 mg/dL (53 to 97.2 µmol/L) for women; ^†^: subjects with normal ALPL value and enrolled after the NGS identified the ALPL variant.
