# Supplementary material for: Clinical and molecular description of the first Italian cohort of 33 subjects with hypophosphatasia
Source: Front Endocrinol (Lausanne). 2023 Aug 1;14:1205977. doi: 10.3389/fendo.2023.1205977 (PMC10433156; doi:10.3389/fendo.2023.1205977)
Supplement: Supplementary file 3 [file Table_3.docx]

**Clinical and molecular description of the first Italian cohort of 33 subjects with Hypophosphatasia**

**Journal of Endocrinological Investigation**

L Cinque, F Pugliese, AS Salcuni, D Trombetta, C Battista, T Biagini, B Augello, G Nardella, F Conti, S Corbetta, R Fischetto, T Foiadelli, A Gaudio, C Giannini, E Grosso, G Guabello, S Massuras, A Palermo, L Politano, F Pigliaru, RM Ruggeri, E Scarano, P Vicchio, S Cannavò, M Celli, F Petrizzelli, M Mastroianno, M Castori, A Scillitani, V Guarnieri

Division of Medical Genetics, Fondazione IRCCS Casa Sollievo della Sofferenza, 71013 Foggia, Italy; v.guarnieri@operapadrepio.it

| **Variant** | **dbSNP, ID** | **gnomAD, MAF** |
| --- | --- | --- |
| c.181+52C>A (het) | rs1767430 | 0.3367 |
| p.(Ser110Ser) | rs1780316 | 0.9449 |
| p.(Leu151Leu) | rs377693871 | 0.00008 (TOPMED) |
| p.(Arg152His) | rs149344982 | 0,011 |
| c.472+8del/insG | rs35423948 | 0.08 (dbSNP) |
| c.473-70A>G | rs1767428 | 0.9871 |
| c.792+45G>T | rs3738098 | 0.1285 |
| p.Tyr263His | rs3200254 | 0.1833 |
| c.793-31C>T | rs1256328 | 0.1588 |
| c.862+20G>T | rs2275377 | 0.1827 |
| c.862+51G>A | rs2275376 | 0.1826 |
| c.862+58C>T | rs2275375 | 0.26 |
| c.863-46G>A | rs74063110 | 0.1793 |
| c.863-12C>G | rs75829132 | 0.1821 |
| c.863-7T>C | rs74063111 | 0.1763 |
| p.(Pro292Pro) | rs3200255 | 0.1848 |
| c.1189+19G>T | rs61778393 | 0.02181 |
| c.1190-65C>A | rs1780329 | 0.2484 |
| c.1309+46C>T | rs4654760 | 0.1270 |
| p.(Ala514Ala) | rs3200256 | 0.00009 |
| p.(Ala460Ala) | rs371984578 | 0.00005795 |
| p.(Val522Ala) | rs34605986 | 0.09373 |

Supplemental Material 3: SNPs with corresponding ID in dbSNP and MAF annotated in gnomAD
